# Rivaroxaban concentrations in acute stroke patients with different dosage forms

**DOI:** 10.1371/journal.pone.0214132

**Published:** 2019-03-21

**Authors:** Shinichi Wada, Manabu Inoue, Takayuki Matsuki, Takuya Okata, Masaya Kumamoto, Naoki Tagawa, Akira Okamoto, Toshiyuki Miyata, Masafumi Ihara, Masatoshi Koga, Kazunori Toyoda

**Affiliations:** 1 Department of Cerebrovascular Medicine, National Cerebral and Cardiovascular Center, Osaka, Japan; 2 Division of Clinical Chemistry, National Cerebral and Cardiovascular Center, Osaka, Japan; 3 Department of Neurology, National Cerebral and Cardiovascular Center, Osaka, Japan; University of Bologna, ITALY

## Abstract

**Background:**

The crushed-tablet rivaroxaban concentration has been previously reported to be lower than the non-crushed concentration. However, the rivaroxaban concentration of fine granules has not yet been investigated. The anticoagulation intensity of rivaroxaban with fine granules, tablets, and crushed tablets was compared in acute stroke patients to assess the efficacy of each form.

**Methods and findings:**

Hospitalized patients over 75 years old with acute stroke who started taking rivaroxaban from April 2012 to September 2017 were included. Blood samples were drawn just before and 4 hours after taking rivaroxaban on a median of 5 days after treatment initiation for concentration measurements (C_0h_, C_4h_) based on an anti-factor Xa chromogenic assay. Of 114 patients (49 female, 83±5 years old), 97 had ischemic strokes, 9 had transient ischemic attacks, and 8 had intracerebral hemorrhages. Rivaroxaban was administered a median of 7 days after onset. Of these, 38 patients were given the 15 mg dose, and 76 were given the 10 mg dose. In the 15 mg dose group, C_0h_ was significantly higher in the fine granule group than in the crushed tablet group, with no significant difference compared to the tablet group [C_0h_: 27.6±6.8 vs 4.0±4.1 (P = 0.01) vs. 33.3±25.2 ng/ml, (P = 0.51), respectively], as was C_4h_ [223.0±66.6 vs 103.0±79.5 (P = 0.02) vs. 229.5±121.6 ng/ml (P = 0.88)]. In the 10 mg dose group, C_0h_ was significantly higher in the fine granule group than in the crushed tablet group and comparable to that in the tablet group [23.2±7.9 vs 7.5±6.2 (P<0.01) vs 19.0±15.8 ng/ml, (P = 0.35)], as was C_4h_ [150.7±85.4 vs 85.1±46.8 (P<0.01) vs 189.8±92.7 ng/ml (P = 0.18)].

**Conclusions:**

The rivaroxaban concentration with fine granules was consistent with that in the tablet group and higher than that in the crushed tablet group.

## Introduction

Recently, direct oral anticoagulants (DOACs) have been widely used in routine practice. Patients treated with DOACs have no need for frequent blood tests, and DOACs have less pharmacological interactions with other drugs[1–4]. DOACs are given to patients for secondary prevention as well, but some patients have difficulties taking DOACs in tablet form, since 22–70% of stroke patients have swallowing disorders[5–7], and some of them develop fatal aspiration pneumonia[8–10]. Unfortunately, they are often given crushed tablets instead of tablets[11], since rivaroxaban, used since April 2012 in Japan, given as crushed tablets has been reported to lead to lower blood concentrations than the usual tablets[12–14]. For these reasons, rivaroxaban fine granules were developed and have recently been used for these patients since December 2015 in Japan. Although clinical trials evaluated rivaroxaban concentrations with fine granules in volunteers before approval[14], no clinical study has compared the rivaroxaban concentrations and examined the safety and efficacy of rivaroxaban in each form. The aim of this study was to measure and clarify the rivaroxaban concentrations and the rates of hemorrhagic and ischemic events with fine granules and compare them with those with tablets and crushed tablets. In addition, only Japan has fine granules of DOACs clinically available, and this was a unique opportunity to investigate the blood concentrations of all three forms.

## Materials and methods

### Participants

This was a single-center, prospective, observational study. Acute ischemic stroke/transient ischemic attack (TIA)/acute intracerebral hemorrhage patients over 75 years of age who were admitted to our center within 7 days after onset and started rivaroxaban for non-valvular atrial fibrillation (NVAF) from April 2012 to September 2017 were included. Ethical approval for this study was obtained from the committee of the National Cerebral and Vascular Center, and written, informed consent was obtained from each patient or the family.

### Procedures

**Fig 1:**
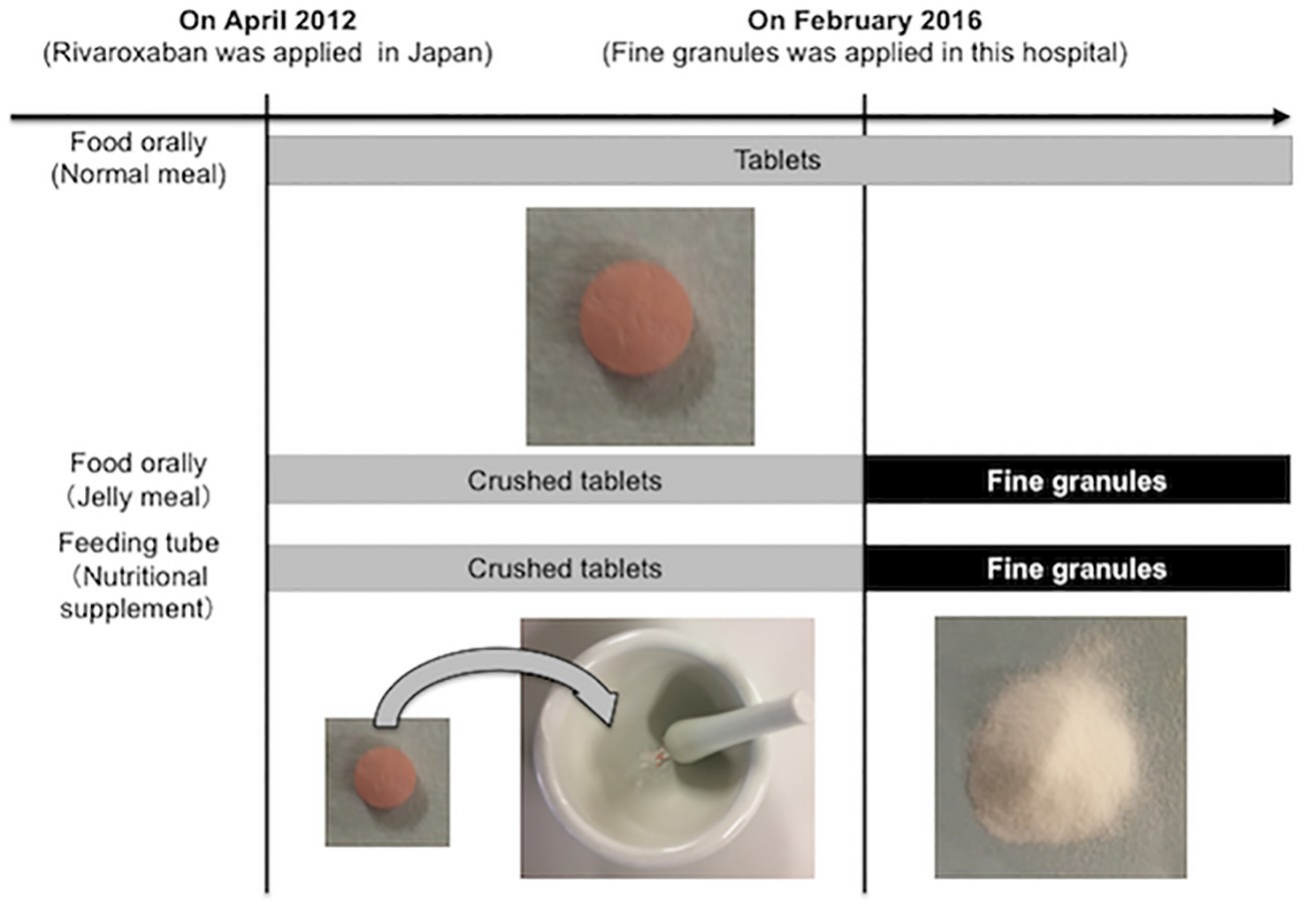
Changes of rivaroxaban forms in this hospital.

Among patients admitted to our cerebrovascular unit due to stroke and TIA, data of patients who had NVAF and were started on rivaroxaban for the prevention of stroke and systemic embolism were collected prospectively. From April 2012 to January 2016, rivaroxaban tablets were given to patients who did not have swallowing disorders, and crushed rivaroxaban tablets were given to patients with swallowing disorders orally or through a feeding tube (Fig 1). From February 2016 to September 2017, rivaroxaban fine granules were given to these patients orally or through a feeding tube instead of crushed tablets. Patients enrolled in this study were assigned to receive either rivaroxaban depending on their characteristics and condition by the attending physicians. In principle, the rivaroxaban dosage was chosen according to the recommended regimen in Japan, which was 15 mg once daily for patients with creatinine clearance (CCr) ≥50 ml/min and 10 mg once daily for patients with 15<CCr<50 ml/min[15,16]. The patients’ baseline characteristics and rivaroxaban concentrations were evaluated retrospectively. The baseline characteristics of the patients, including the CHADS_2_ score and the HAS-BLED score, weight, renal function, and other medications, on the day of blood collection were recorded[17–19]. Renal function was defined as CCr using the Cockcroft and Gault equation.

Blood sampling was performed at least 2 days after rivaroxaban was started to evaluate the steady-state concentration. Two venous blood samples were collected each time in citrate-containing tubes just before (trough) and 4 h (peak) after drug administration. The sampling point at 4 h was meant to capture the maximum rivaroxaban concentration, because the maximum concentration has been reported to occur 1 to 3 hours after tablet intake and to be delayed by 2 h with diet[12]. For 1 of the 2 tubes, following double centrifugation at 2500 g for 15 min, platelet-poor plasma was collected, quick-frozen, and stored at -80°C until the analysis for rivaroxaban concentration was performed. The concentration was measured at the trough and peak points (anti-factor Xa chromogenic assay, STA-Liquid Anti-Xa; Diagnostica Stago, Asnieres, France).

### Outcome

The primary outcome measures were the concentrations in the fine granule group compared to those in the tablet group and the crushed tablets group. The secondary outcome measures were the rates of hemorrhagic and ischemic events in the groups. Hemorrhagic events were defined as major hemorrhagic events according to the classification of the International Society on Thrombosis and Hemostasis[20]. Ischemic events were defined as recurrence of ischemic stroke or TIA, categorized according to the Trial of Org 10172 in Acute Stroke Treatment (TOAST) classification[21], and systemic embolism, acute coronary syndrome, aortic dissection, aortic aneurysm rupture, peripheral artery disease requiring hospitalization, venous thromboembolism, and revascularization such as carotid endarterectomy, carotid artery stenting, and percutaneous coronary intervention[22]. Events were ascertained at outpatient clinics (or by telephone survey) for as long as possible.

### Statistical analysis

Baseline characteristics and rivaroxaban concentrations in all three forms were analyzed by Student’s *t*-test and the chi-squared test. All data were analyzed with JMP version 12.0.1 (SAS Institute, Cary, NC, USA), and the level of significance was set as P<0.05 (two-tailed).

## Results

**Fig 2:**
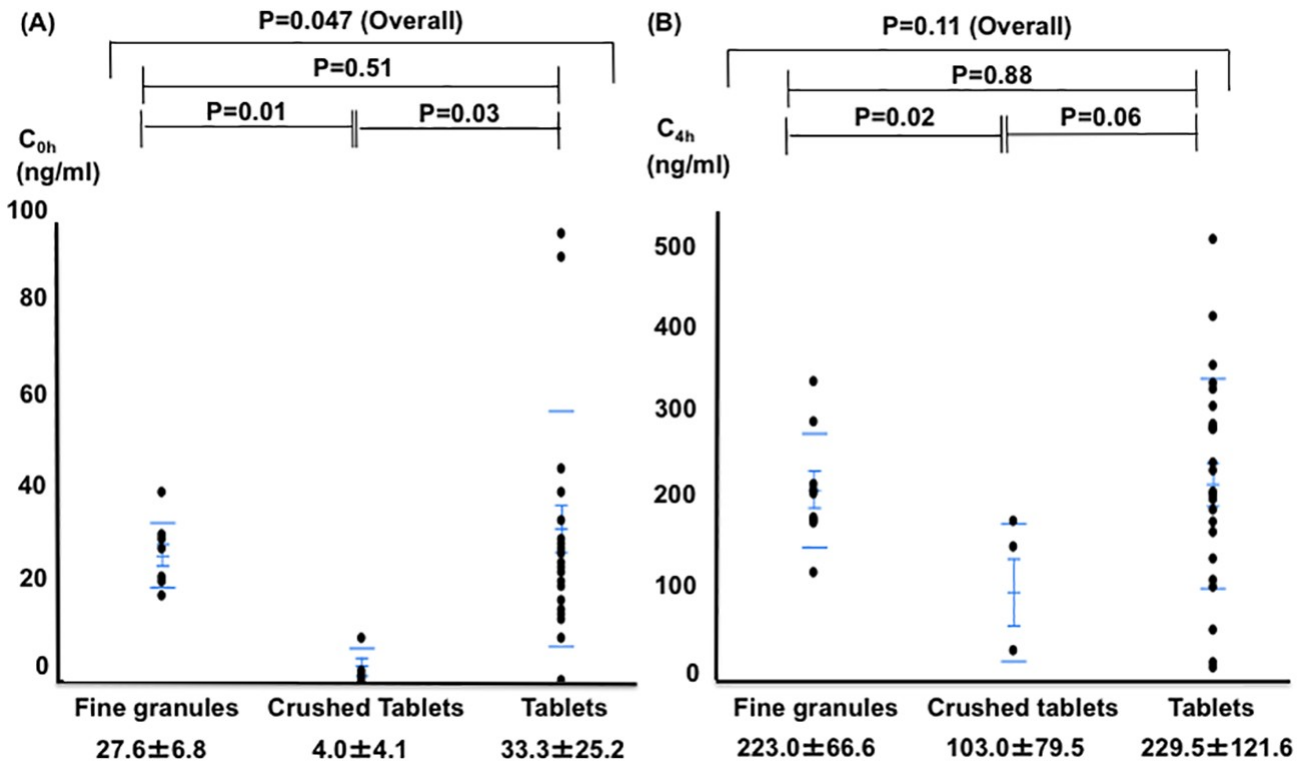
Comparison of rivaroxaban concentrations at 15 mg daily in the three groups. (A) At the trough point. (B) At the peak point.

Of 114 patients (49 female, 83±5 years old), 97 had ischemic strokes, 9 had TIAs, and 8 had intracerebral hemorrhages, with rivaroxaban administered a median of 7 days after onset. Of these, 38 patients were given the 15 mg dose, and 76 were given the 10 mg dose. In the 15 mg daily group, patients who took fine granules (n = 9) and crushed tablets (n = 4) had higher NIHSS scores than those who took tablets (n = 25) (Table 1). The trough concentration (C_0h_) was significantly higher in the fine granule group than in the crushed tablet group, with no significant difference compared to the tablet group [27.6±6.8 vs 4.0±4.1 (P = 0.01) vs. 33.3±25.2 ng/ml, (P = 0.51), respectively] (Fig 2). The peak concentration (C_4h_) was also higher in the fine granule group than in the crushed tablet group and equivalent to that in the tablet group [223.0±66.6 vs 103.0±79.5 (P = 0.02) vs. 229.5±121.6 ng/ml (P = 0.88), respectively].

**Table 1:** Baseline characteristics with rivaroxaban 15 mg daily.

	Fine granules n = 9	Crushed tablets n = 4	Tablets n = 25
Female	1 (11)	2 (50)	11 (44)
Age, y	82±4	80±4	80±3
Congestive heart failure	0	0	1 (4)
Hypertension	6 (67)	3 (75)	19 (76)
Diabetes mellitus	2 (22)	1 (25)	10 (40)
Stroke on admission			
Ischemic stroke	9 (100)	3 (75)	20 (80)
Transient ischemic attack	0	0	4 (16)
Intracerebral hemorrhage	0	1 (25)	1 (4)
Old ischemic stroke	2 (22)	0	5 (20)
Warfarin on admission	0 *	3 (75)	9 (36)
DOACs on admission†	0	0	4 (16)
CHADS_2_ score‡	2 [1–4]	2 [1–3]	2 [2–3]
HAS-BLED score‡	3 [2–3]	2 [1–3]	2 [2–3]
Weight, kg	54.5±7.4	59.9±13.6	59.2±7.9
NIHSS score on admission§	18 [3–27] *	21 [6–24] *	4 [2–11]
Creatinine clearance, ml/min ||	54.1±9.1	64.8±14.5	63.6±13.2
Nasal tube	5 (56)	4 (100)	-

n (%) or average ± standard deviation or median [interquartile range]

* P value<0.05 compared to tablets.

^†^ DOACs indicates direct oral anticoagulants including dabigatran, rivaroxaban, apixaban, or edoxaban.

^‡^ CHADS_2_ score and HAS-BLED score were calculated from data before onset.

^§^ NIHSS: National Institutes of Health Stroke Scale

^||^ Creatinine clearance was calculated from the Cockcroft-Gault equation.

**Fig 3:**
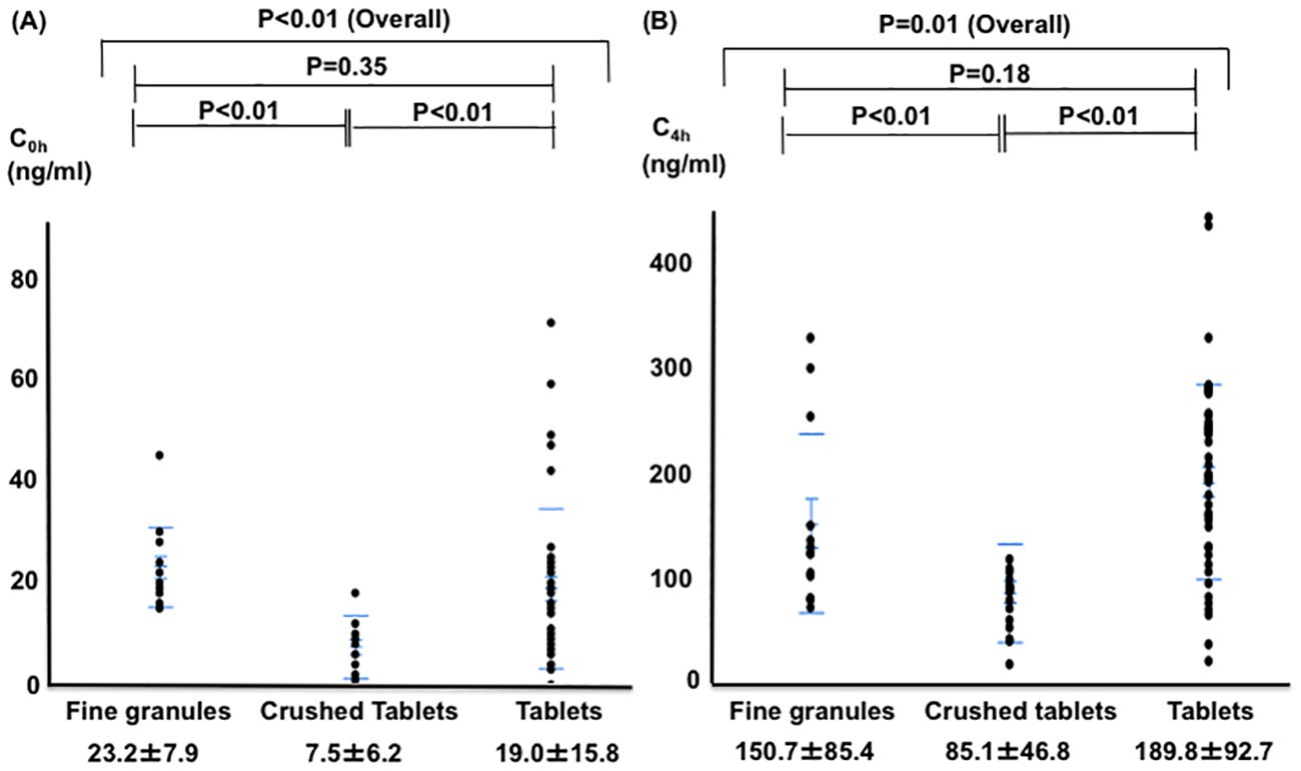
Comparison of rivaroxaban concentrations at 10 mg daily in the three groups. (A) At the trough point. (B) At the peak point.

In the 10 mg daily group, patients in the fine granule (n = 13) and crushed tablet (n = 19) groups had significantly higher NIHSS scores, and patients in the crushed tablet group had significantly lower weight compared to the tablet group (n = 44) (Table 2). C_0h_ was significantly higher in the fine granule group than in the crushed tablet group and comparable to that in the tablet group [23.2±7.9 vs 7.5±6.2 (P<0.01) vs 19.0±15.8 ng/ml, (P = 0.35)], as was C_4h_. [150.7±85.4 vs 85.1±46.8 (P<0.01) vs 189.8±92.7 ng/ml (P = 0.18)] (Fig 3).

**Table 2:** Baseline characteristics with rivaroxaban 10 mg daily.

	Fine granules n = 13	Crushed tablets n = 19	Tablets n = 44
Female	7 (54)	9 (47)	19 (43)
Age, y.	86±8	84±5	83±5
Congestive heart failure	5 (38)	8 (42) *	7 (16)
Hypertension	10 (77)	16 (84)	32 (73)
Diabetes mellitus	4 (31)	6 (32)	12 (27)
Stroke on admission			
Ischemic stroke	13 (100)	16 (84)	36 (82)
Transient ischemic attack	0	0	5 (11)
Intracerebral hemorrhage	0	3 (16)	3 (7)
Old ischemic stroke	2 (15)	2 (11)	9 (20)
Warfarin on admission	3 (23)	9 (47)	13 (30)
DOACs on admission†	4 (31)	1 (5)	4 (9)
CHADS_2_ score‡	3 [2–4]	3 [2–4]	2 [2–3]
HAS-BLED score‡	2 [2–3]	2 [2–3]	2 [2–3]
Weight, kg	49.8±9.3	49.2±8.0 *	55.6±11.2
NIHSS score on admission§	20 [16–26] *	19 [11–24] *	4 [2–12]
Creatinine clearance, ml/min ||	42.5±15.8	41.7±11.9	47.7±15.5
Nasal tube	8 (62)	12 (63)	-

n (%) or average ± standard deviation or median [interquartile range]

* P value<0.05 compared to tablets.

^†^ DOACs indicates direct oral anticoagulants including dabigatran, rivaroxaban, apixaban, or edoxaban.

^‡^ CHADS_2_ score and HAS-BLED score were calculated from data before onset.

^§^ NIHSS: National Institutes of Health Stroke Scale

^||^ Creatinine clearance was calculated from the Cockcroft-Gault equation.

In the fine granule group, the concentrations tended to be lower in patients who took the medication through a feeding tube compared to patients who took it orally in the 15 mg daily group (C_0h_, C_4h_: 27.4±4.6, 188.6±38.4 ng/ml vs 27.8±9.8, 266.0±73.7 ng/ml) and in the 10 mg daily group (C_0h_, C_4h_:20.8±3.8, 118.8±59.7 ng/ml vs, 27.2±11.3, 201.8±101.7 ng/ml) (S1 Fig).

S1 Table shows the ischemic or hemorrhagic events that occurred in the patients who started rivaroxaban in this study. Follow-up periods were 27 [interquartile range (IQR) 9–67] days in the fine granule group, 89 (IQR 34–518) days in the crushed tablet group, and 189 (IQR 22–550) days in the tablet group. Of 7 ischemic stroke patients, four patients were administered rivaroxaban through a feeding tube. In the fine granule group, two patients developed recurrent ischemic stroke after starting rivaroxaban; one had an ischemic stroke classified into other stroke determined by the TOAST classification and caused by Trousseau syndrome, and the other had ischemic stroke classified as large artery disease. With regard to hemorrhagic events, two patients developed musculoskeletal hemorrhage, one patient in the fine granule group and the other in the tablet group.

## Discussion

In this study, rivaroxaban concentrations were assessed in acute stroke patients by dosage form. The concentration in the fine granule group was higher than in the crushed tablet group and comparable to that in the tablet group in the present study.

We recently reported that administering rivaroxaban as crushed tablets led to reduced plasma concentrations. Loss of rivaroxaban in the process of tablet crushing, occlusion in the feeding tube, and decreased absorption by direct administration to the small bowel may contribute to the lowering of rivaroxaban concentrations[12,23,24]. The present results suggest that fine granules were effective in maintaining the same degree of concentration as tablets, as shown in the pharmacological interview form regardless of the slight difference in baseline characteristics[14]. Even so, the plasma concentrations for patients who took fine granules through a feeding tube tended to be lower than those of patients who took rivaroxaban orally. This result indicates that active ingredient loss in the process of tablet crushing was reduced, but the problem of occlusion in the feeding tube or decreased absorption in the small bowel may still remain, but to a lesser extent.

The outcomes for this study were all hemorrhagic and ischemic events, with follow-up for as long as possible. As for ischemic events, although two patients in the fine granule group developed ischemic strokes in the early stage after starting rivaroxaban, there was no recurrence of cardioembolic stroke. In both cases in the fine granule group, the drug was administered by feeding tube, and the concentration tended to decrease. Although given in fine granule form with minimum loss of the whole dose, administration through the feeding tube tended to cause blood concentration lowering, and this may have led to ischemic events. Given these findings, administration through a feeding tube must be performed with great caution.

There are several limitations in this study. First, the sample size was not large enough, and there was a divergence between the numbers of the fine granule group and the other groups. Patients were not randomized to a formulation, because the physician determined the formulation. Since the dose and administration rates of feeding enteric nutrients through the feeding tube and the gastric position of the feeding tube were also slightly different among patients, these differences may have affected the concentrations. Moreover, strictly, multivariate analysis by factors with significant differences in baseline characteristic should have been done for blood concentrations, but the analysis could not be performed because of the small sample size. However, since the main factors that can affect the blood concentration, such as age and CCr, did not generally show significant differences, the effect is considered small. Second, some studies noted that blood collection at 4 hours after taking DOACs does not always reflect the peak concentration. Some reports suggested that nasogastric tube feeding will cause a double peak in concentration due to differences in sizes of the crushed drug particles, the transit time associated with a liquid meal while digesting, or a too deeply placed feeding tube itself that might bypass the pyloric sphincter and pour medication directly into the duodenum[13]. Third, the important limitation is that the follow-up periods in patients with fine granules were too short, and it was not possible to statistically assess the clinical outcomes and complications for at least 3 months after onset, such as recurrence of ischemic stroke or hemorrhage events. It has been reported that more patients suffered from hemorrhagic events in clinical practice than in randomized trials[25]. Further examination is needed to establish the safety of fine granules, because the usage of fine granules may also have an effect on the rates of hemorrhagic events in clinical practice.

The reason for these limitations is that rivaroxaban fine granules have not been available until recently, and the fine granules were not used widely even in Japan. Rivaroxaban fine granules are not used in many rehabilitation hospitals in Japan due to the high cost, although rivaroxaban may decrease NVAF events and decrease overall medical costs in Japan [26]. Another reason is that, in rehabilitation hospitals, anticoagulants for patients with NVAF may be switched to warfarin or rivaroxaban with crushed tablets or to tablets when swallowing disorders improved.

In common practice, tablet crushing is generally not recommended for patients with swallowing disorders. Sometimes, it can result in any of a fatal overdose, under-dosing, or ineffective treatment[27]. From the perspective of the concentration, it is expected that rivaroxaban fine granules have efficacy or safety equivalent to tablets among acute stroke patients with swallowing disorders. The need to administer rivaroxaban by fine granules to acute stroke patients with swallowing disorders would increase, since some recent studies proved that early initiation of Xa inhibitors was generally safe[21,28]. The findings of the present paper may influence the treatment strategy for acute stroke and other rehabilitation facilities, and rivaroxaban should be administered with the option of all three forms depending on each patient’s level of dysphagia to ensure the optimal clinical outcomes. Furthermore, a randomized clinical trial is needed to demonstrate the effectiveness of the administration of fine granules for acute stroke patients.

## Conclusions

Concentrations of rivaroxaban given as fine granules were assessed in patients with acute ischemic/hemorrhagic stroke. Use of fine granules allows plasma concentrations similar to those of tablets to be maintained, unlike with crushed tablets.

## Supporting information

S1 Fig Comparison of rivaroxaban plasma concentrations in patients who took 15 mg or 10 mg daily by fine granules between oral and feeding tube administration. (A) At the trough point in the 15 mg daily group. (B) At the peak point in the 15 mg daily group. (C) At the trough point in the 10 mg daily group. (D) At the peak point in the 10 mg daily group.
(TIFF)

S1 Table Ischemic events and major hemorrhagic events after starting rivaroxaban (fine granules or crushed tablets, or tablets). (DOCX)
