# Supplementary figures and images for: Rivaroxaban concentrations in acute stroke patients with different dosage forms
Source: PLoS One. 2019 Mar 21;14(3):e0214132. doi: 10.1371/journal.pone.0214132 (PMC6428291; doi:10.1371/journal.pone.0214132)

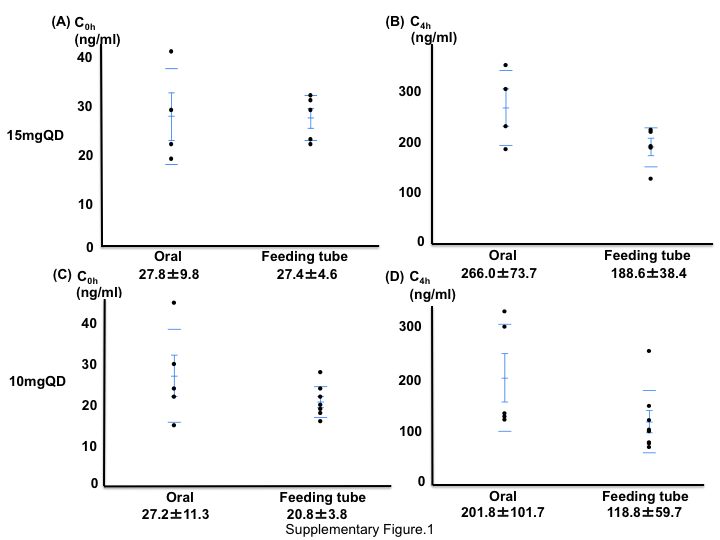

Supplement: S1 Fig — (A) At the trough point in the 15 mg daily group. (B) At the peak point in the 15 mg daily group. (C) At the trough point in the 10 mg daily group. (D) At the peak point in the 10 mg daily group. (TIFF) [file pone.0214132.s001.tiff]
