# Supplementary material for: Rivaroxaban concentrations in acute stroke patients with different dosage forms
Source: PLoS One. 2019 Mar 21;14(3):e0214132. doi: 10.1371/journal.pone.0214132 (PMC6428291; doi:10.1371/journal.pone.0214132)
Supplement: S1 Table — (DOCX) [file pone.0214132.s002.docx]

**Table S1. Ischemic events and major hemorrhagic events after starting rivaroxaban (fine granules, crushed tablets, or tablets)**

| case |  | Dosage form/  dose route | Dose | Diagnosis on admission† | C_0h_, ng/ml | C_4h_, ng/ml | Weight, kg | CCr,  ml/min‡ | CHADS_2_  score§ | HAS-BLED  score§ | Time from starting to event, days | Events after taking rivaroxaban\|\| |
| --- | --- | --- | --- | --- | --- | --- | --- | --- | --- | --- | --- | --- |
| Ischemic events | | | | | | | | | | | | |
| 1 |  | Fine/Tube | 10 | IS | 19 | 121 | 49 | 43 | 2 | 2 | 7 | IS (OD) |
| 2 |  | Fine/Tube | 10 | IS | 28 | 100 | 42 | 37 | 3 | 2 | 3 | IS (LAD) |
| 3 |  | Crushed/Tube | 15 | ICH | 2 | 35 | 57 | 60 | 1 | 1 | 23 | VTE (DVT) |
| 4 |  | Crushed/Tube | 15 | IS | 10 | 186 | 60 | 68 | 2 | 2 | 138 | VTE (DVT) |
| 5 |  | Tablet | 15 | IS | 91 | 515 | 63 | 63 | 5 | 3 | 16 | IS (CE) |
| 6 |  | Tablet | 10 | TIA | 22 | 68 | 74 | 75 | 2 | 2 | 82 | IS (SVD) |
| 7 |  | Tablet | 10 | IS | 11 | 282 | 65 | 44 | 4 | 3 | 431 | IS (CE) |
| Major hemorrhagic events | | | | | | | | | | | | |
| 8 |  | Tablet | 10 | IS | 6 | 93 | 59 | 60 | 4 | 3 | 68 | Musculoskeletal hemorrhage |
| 9 |  | Fine/Oral | 10 | IS | 24 | 298 | 45 | 86 | 1 | 1 | 6 | Musculoskeletal hemorrhage |

† IS: ischemic stroke，TIA: transient ischemic attack, ICH: intracerebral hemorrhage

‡ Creatinine clearance was calculated from the Cockcroft-Gault equation.

§ CHADS_2_ score and HAS-BLED score were calculated from data before onset.

|| IS: ischemic stroke, LAD: large artery disease, CE: cardioembolic stroke, SVD: small vessel disease, OD: stroke of other determined etiologies, VTE: venous thromboembolism, DVT: deep vein thrombosis
